# A new technique, combined plication-incision (CPI), for correction of penile curvature

**DOI:** 10.1590/S1677-5538.IBJU.2016.0578

**Published:** 2018

**Authors:** Hamed Abdalla Hamed, Mohamed Roaiah, Ahmed M. Hassanin, Adham Ashraf Zaazaa, Mahmoud Fawzi

**Affiliations:** 1 Department of Andrology, Faculty of Medicine, Cairo University, Cairo, Egypt

**Keywords:** Penile Induration, Penis, Erectile Dysfunction

## Abstract

**Introduction:**

Penile curvature (PC) can be surgically corrected by either corporoplasty or plication techniques. These techniques can be complicated by post-operative: penile shortening, recurrent PC, painful/palpable suture knots and erectile dysfunction.

**Objective:**

To avoid the complications of corporoplasty and plication techniques using a new technique: combined plication-incision (CPI).

**Materials and Methods:**

Two groups (1&2) were operated upon: group 1 using CPI and group 2 using the 16-dot technique. In CPI, dots were first marked as in 16 dot technique. In each group of 4 dots the superficial layer of tunica albuginea was transversely incised (3-6mm) at the first and last dots. Ethibond 2/0, passed through the interior edge of the first incision plicating the intermediate 2 dots and passed out of the interior edge of the last incision, was tightened and ligated. Vicryle 4/0, passed through the exterior edges of the incisions, was tightened and ligated to cover the ethibond knot.

**Results:**

Twelve (57.1 %) participants in group 2 complained of a bothering palpable knot compared to none in group 1 with statistically significant difference (P=0.005). Postoperative shortening (5mm) of erect penis, encountered in 9 participants, was doubled in group 2 but with insignificant difference (P>0.05). Post-operative recurrence of PC, was encountered in only 1 (4.8%) participant in group 2, compared to none in group 1, with insignificant difference (P>0.05). Post-operative erectile rigidity was normally maintained in all participants.

**Conclusion:**

The new technique was superior to the 16-dot technique for correction of PC.

## INTRODUCTION

Penile curvature (PC) is not an uncommon disorder in men. PC may be congenital or acquired, mostly caused by Peyronie's disease (1). PC may cause difficulty and pain during intercourse, or even coital impairment, and severe psychological problems due to the cosmetic appearance of the affected person (2,3). Surgical correction of the penile curvature is required when coital function is impaired (3).

Surgical correction of PC can be classified into three main categories: tunical shortening procedures, tunical lengthening procedures, and penile prosthesis implantation (4). Tunical shortening is the commonly used surgical technique for correction of PC. Tunical shortening restricts the convex, longer, side of the penis to match the length of the oppositely shorter side (5).

Tunical shortening procedures include either incisional/excisional corporoplasty techniques or non-incisional plication techniques (6). In corporoplasty, a tunical ellipse is excised or single (or multiple) longitudinal tunical incision(s) is (are) made and the remaining defect is closed horizontally (7–9). Plication of the tunica includes Essed & Schröder technique (10) and 16 dot technique (11).

Tunical shortening complications include loss of length, recurrent or residual penile curvature, erectile dysfunction, change in penile sensation, and painful or palpable suture knots. Many of these outcomes can be quite distressing to the patient (5).

In this study, we describe a new technique, where we combine the plication and the incisional techniques, without opening of the cavernous tissue, aiming to get the advantages and to avoid the disadvantages of either technique.

## MATERIALS AND METHODS

This study was designed to compare a new technique: combined plication-incision (CPI), with the 16 dot technique for correction of penile curvature. The study was approved by the Andrology board and Ethics Committee, Faculty of Medicine, Cairo University.

In the period from Dec 2013 till Oct 2015 one hundred ninety men presented with a complaint of penile curvature to the Andrology outpatient clinic at the University hospital. Seventy nine cases required surgery for correction of curvature and matched the inclusion and exclusion criteria. Thirty nine eligible participants agreed to participate in this research and to be available for follow-up. Informed consents were taken from participants in the study.

For potentially eligible participants of men presenting with a complaint of penile curvature, the following procedures were designed: history taking, physical examination, international index of erectile function questioning, intra-corporeal injection and penile duplex.

History taking included: age, history of general diseases (e.g. diabetes mellitus, hypertension), Dupuytren's contracture, history of any accidents/genital trauma and medications as beta-blocker and Methotrexate. Any history of psychiatric disease and/or treatment was reported.

Sexual history included the presence of normal erectile rigidity, easy intromission, presence/absence of painful erection, and/or painful intercourse to any partner. The patient was also asked about desire, orgasm and ejaculatory problems. Participants were asked to complete an abridged five-item version of the International Index of Erectile Function (IIEF) (12). During physical examination the penis was inspected for the size and the site of the urethral meatus and was palpated for tenderness, chordae or plaques. An intracorporal injection of 20 micrograms PGE1 was given and the grade of erection was evaluated (13). During rigid erection (E4/E5) the penile length (Table-2) and the angle of penile deviation were measured, by the doctor, using metal seizer and protractor respectively.

Duplex evaluation: peak systolic velocity (PSV) >30cm/min., end diastolic velocity (EDV)<5 cm/min and arterial dilatation more than 70% were considered normal hemodynamics.

### 

#### Inclusion criteria

Men aged >21 years presenting with congenital or acquired penile curvature of >30°, who had normal erectile function, E4 or E5 response on intracorporal injection, and normal penile duplex parameters with stable course in case of Peyronie's disease (more than one year).

#### Exclusion criteria

Men with hypospadias with chordee, epispadias, poor response to PGE1 (response <E4) and/or active phase of Peyronie's disease (less than one year). Laboratory testing (blood glucose, lipid profile and routine preoperative labs) were done for included participants.

#### Selection of patients for either technique

Two groups of patients were designed according to which technique was more suitable for the candidate according to the opinion of the surgeon. The combined plication-incision (CPI) technique was avoided in 2 situations (in which 16-dot technique was preferred): ventral curvature (to prevent mobilization of the neurovascular bundle that may cause postoperative pain) and Peyronie's disease (performing incisions in patients with Peyronie's disease may exaggerate the condition) CPI technique was used mainly for patients with congenital penile lateral or dorsal deviation.

In case with congenital ventral curvature: performing tunical incisions was avoided to preserve structures of neurovascular bundle. However, in four patients only, removal of a segment of the deep dorsal vein allowed a space to perform adequate incisions to use the new technique. Remaining patients with congenital ventral curvature together with acquired cases were operated upon using the 16 dot technique (11) and were named group 2. Participants operated upon by the CPI technique were named group 1.

#### Surgical technique

All participants received spinal anaesthesia and lied in supine position. After scrubbing and draping, an artificial erection was induced by intra-corporeal injection of 20 micrograms of PGE1. Skin incision was made at the circumcision line. Blunt dissection was performed to enter Colle's fascia. The entire penis was then degloved till the level of penile base. Buck's fascia was dissected longitudinally from the coronal sulcus to the base of the penis until tunica albuginea and neurovascular bundle were visualized and identified. Strict care was employed to preserve all structures of neurovascular bundle dorsally and the corpus spongiosum/urethra ventrally. In all cases, the point of maximum curvature was identified during full rigid erection.

#### CPI technique

**Figure 1:**
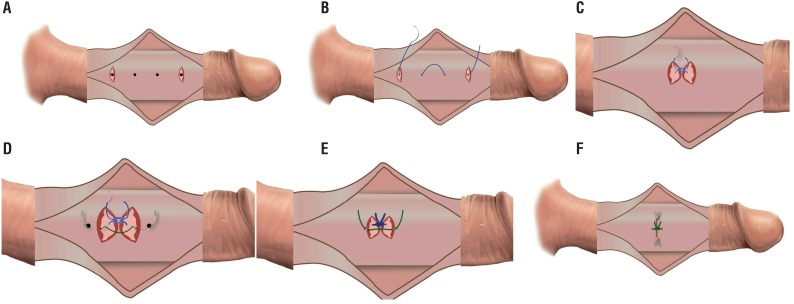
Diagrammatic steps of CPI technique 1A) Two transverse incisions through outer layer of tunica albuginea at first and last dots. 1B and C) Ethibond suture passing through interior edges of the incisions and plicating the intermediate 2 dots. 1D) Vicryl suture passing through outer free edges of the incisions. 1E) Tightening and ligation of the Ethibond. 1F) Closure of outer edges over ethibond suture knot.

**Figure 2:**
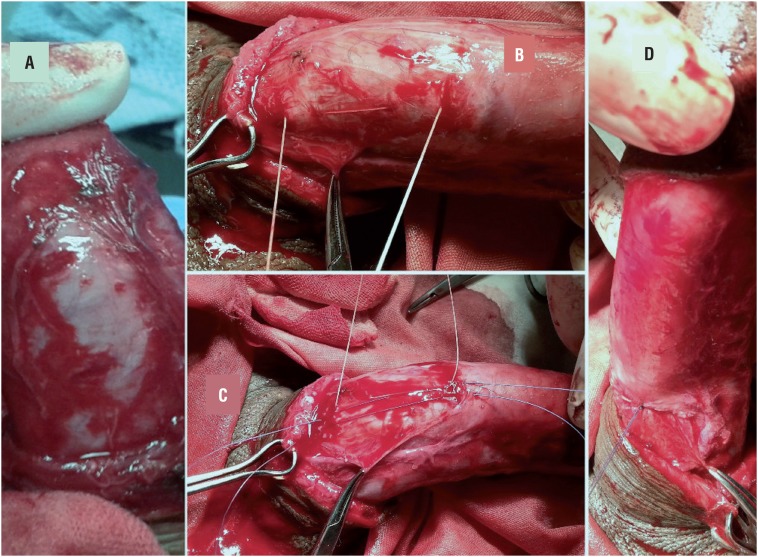
Steps of CPI technique. 2A) Two transverse incisions through outer layer of tunica albuginea at first and last dots. 2B) Ethibond suture passing through interior edges of the incisions and plicating the intermediate 2 dots. 2C) Vicryl suture passing through outer free edges of the incisions. 2D) Tightening and ligation of the Ethibond sutures followed by closure of outer edges over ethibond suture knot.

Sutures were applied on the convex side of the penis just proximal to the point of maximal curvature passing through 8 dots. Those 8 dots were divided into two lines of 5mm spaced 4 dots, on either side of the middle line “in cases of ventral/dorsal deviation” and parallel to each other on right or left side in cases with lateral deviation. Transverse incisions 3-6mm were performed, with a fine scalpel, at the site of the first and last dots (of each group of 4 dots) through the superficial layer of tunica albuginea (Figures 1A and 2A), without cutting into the corpus cavernosum, to prepare a cavity for the knots. The 00 ethibond suture was passed through the interior edge of the first incision, plicating the intermediate 2 dots to end out from the interior edge of the last incision (Figures 1B and 2B), leaving free exterior incisions edges (Figure-1C). Vicryl 4/0 suture was passed through the exterior edges of the incisions (Figures 1D and 2C). Tightening and ligation of the ethibond was first done (Figure-1E) and the vicryl was then tightened and ligated over the ethibond suture knot to cover it (Figures 1F and 2D).

Sixteen dot technique was performed according to the description of Gholami and Lue (11).

In all cases, for proper correction during tightening of ethibond sutures, the penis should not be in full rigidity and the assistant should bend the distal shaft in the direction of correction to allow proper tightening of the suture. Avoidance of crushing of the suture material with instruments during ligation is mandatory.

After ligation of the 1^st^ set of sutures a full rigid erection was induced to assess straightness of the penis. Usually there was a remaining deviation but with point of maximum deviation displaced distally. The previous steps were repeated till reaching a complete straightness. Over-correction was avoided by measuring the degree of correction before ligating the sutures. This was done by traction of the two suture ends against each other simulating ligation. By inspecting the degree of correction we decided the degree of tightening of the suture needed whether completely tight or little looser. The second plane of penile curvature was corrected using the same technique used for correction of the main plane.

Buck's and superficial fascia were closed longitudinally with continuous vicryl 3/0 or 4/0 sutures. Postoperative erected penile length was measured (Table-2) and rigidity was evaluated. Skin was closed with vicryl 5/0 in continuous manner but in three separate parts. Then, dressing was applied with minimal pressure on the penile shaft for bandage. Finally, 1cc of diluted phenylephrine was injected intracorporeally if no spontaneous detumescence occurred.

#### Follow up

A weekly postoperative follow up was done to ensure complete wound healing and to manage any post-operative complications if present. After 1 month, measurement of the postoperative flaccid length was performed.

Examination for palpable suture knot along the shaft was performed by a doctor who was blind to the operative procedure (patients’ group). Furthermore, the patient was questioned about sensation of a knot and if it bothers him. However, we decided to rely on the patient words for assortment of cases positive for palpable suture knot. After 3 months, after resuming sexual activity, participants were followed up by the IIEF-5. Furthermore, all participants were asked about any decrease in the erectile function noticed and about a recurrence of deviation. This was repeated every 3 months till the end of follow-up period that ranged from 12 to 31 months with mean a duration of 20.5±6.9 months.

### Statistical analysis

Data were statistically described in terms of range, frequencies, percentages, mean and standard deviation (SD). Comparison used Student t test, Mann Whitney U test, paired t test and Wilcoxon signed rank test. Correlation between various variables was done using Spearman Rank correlation equation. For comparing categorical data, Chi square test was performed. Exact test was used instead when the expected frequency was less than 5. P values <0.05 were set as statistically significant. All statistical calculations were done using computer program SPSS (Statistical Package for the Social Science; SPSS Inc., Chicago, IL, USA) release 15 for Microsoft Windows (2006).

## RESULTS

This study included 39 participants, 18 in group 1 and 21 in group 2. Mean age was 30.6±10.2, 28±7.3 and 32.8±11.8 for all participants, group 1 and group 2 respectively with insignificant difference (P=0.14). Sixteen (41%) participants were smokers, 2 (5.1%) were hypertensive and 1 (2.6%) was diabetic type 2 with insignificant difference between both groups regarding these data (P>0.05). Five (12.8%) participants had acquired curvature for more than 1.5 year. All of them were assigned to group 2 to avoid exaggeration of the condition with incisions.

Thirty one (79.5%) participants had single plane deviation and eight (20.5%) had bidirectional deviation, 3 (16.7%) in group 1 and 5 (23.8%) in group 2. Table-1 presents the preoperative direction of curvature. Deviation angle ranged 30-90 at the main plane and 30-35 at the second plane. The mean of the angles of the main plane was 43.3±18.1 and 53.1±18.1 for group 1 and 2 respectively with insignificant difference (P=0.1).

**Table 1:** Direction of curvature preoperatively among both groups.

	Main plane			Second plane		
	Group 1	Group 2	Total	Group 1	Group 2	Total
**ventral**	4 (22.2%)	13 (61.9%)	17 (43.6%)	0	1 (4.8%)	1 (2.6%)
**Dorsal**	2 (11.1 %)	3 (14.3 %)	5 (12.8%)	0	1 (4.8%)	1 (2.6%)
**Right**	4 (22.2 %)	1 (4.8 %)	5 (12.8 %)	1 (5.6%)	1 (4.8%)	2 (5.1%)
**Left**	8 (44.4%)	4 (19.0%)	12 (30.8%)	2 (11.1%)	2 (9.5%)	4 (10.3%)
**None**	0	0	0	15 (83.3)	16 (76.2)	31 (79.5%)
**Total**	18 (100%)	21 (100%)	39 (100%)	18 (100%)	21(100%)	39 (100%)

**Table 2:** Pre and postoperative flaccid and erected penile length.

	Flaccid penile length (preoperative)		Erected penile length (preoperative)		Erected penile length (postoperative)		Flaccid penile length (postoperative)	
	Range (cm)	Mean±SD	Range (cm)	Mean±SD	Range (cm)	Mean±SD	Range (cm)	Mean±SD
Group 1	10–14	12±1.1	12–18	15.8±1.6	12–18	15.8±1.6	10–14	12±1.1
Group 2	10.5–17	12.4±1.7	13–20	14.9±1.7	12.5–20	14.7±1.7	10.5–17	12.4±1.7
Total	10–17	12.2±1.5	12–20	15.3±1.7	12–20	15.1±1.7	10–17	12.2±1.5

The number of dots marked during the surgeries ranged from 8-24 with a mean 15.28±5.79, 13.33±5.48 and 16.95±5.64 dots, for the total cases, group 1 and group 2 respectively. Significant positive correlation was found between the number of dots and the degree of curvature (r=0.69 and P<0.001).

Post-operative follow up ranged from 12 to 31 months with a mean duration 20.5±6.9, 20.1±7.7 and 20.9±6.4 months for all cases, group 1 and group 2 respectively.

One participant in the group 1 developed a hematoma. Post-operative pain was reported by 3 cases in each group representing 14.3% and 16.7% of group 1 and 2 respectively with insignificant difference (P=0.53). Postoperative shortening was encountered in nine (23.1%), three (16.7%) in group 1 and 6 (28.6%) in group 2 with insignificant difference (P=0.4). Shortening was 0.5cm of the erected penile length in all cases. Twelve (57.1%) participants in group 2 complained of feeling a bothering palpable knot, compared with none in group 1, with statistically significant difference (P=0.005). By examination, palpable knot was found in 16 (76.2%) cases in group 2 and none in group 1. However, we considered cases positive only when reported by the patients themselves after asking about it.

Residual curvature did not exceed 10 degrees. This was encountered in 2 (11.1%) in group 1 and 1 (4.8%) in group 2. Post-operative recurrence of curvature was recorded in only 1 (4.8%) participant in group 2 at the 18^th^ month of his follow-up, compared to none in group 1, with insignificant difference (P=0.586).

Throughout the follow-up period, all participants retained a postoperative rigid erection and none of them reported a postoperative decrease of erectile rigidity. The pre and postoperative IIEF-5 score(s) were reported by 25 (64.1%) participants. The preoperative IIEF-5 score was 20.28±1.41, 20.55±1.38 and 20.04±1.43 for all participants, group 1 and group 2 respectively. The postoperative IIEF-5 was 20.25±1.37, 20.5±1.29 and 20.04±1.43 for all participants, group 1 and group 2 respectively. Comparative studies of the pre and postoperative scores for each group were statistically insignificant (P=0.579 and 1 for group 1 and 2 respectively).

## DISCUSSION

Tunical corporoplasty and tunical plication are two techniques used to correct PC. In tunical corporoplasty, permanent fusion of the tunical margins, by the healing process, adds to the strength of the sutures and allow for much better results in terms of recurrence. However, the invasiveness of corporoplasty is great and the tourniquet, if used for long time, may be harmful to the sensory nerves and the erectile tissue (14). On the contrary, tunical plication procedures are less invasive (15–17) and separation of dorsal neuro-vascular bundles from the tunica albuginea is not done in 16 dot plication technique (11, 18). However, in plication, the strength depends only on the sutures and not on the healing process (11, 17, 19, 20). After tunical plication, the recurrences rates were high (10, 16, 19) and the presence of permanent palpable knots at the site of the tunical sutures (15, 21) was noted causing discomfort or even pain (11, 22, 23). Furthermore, creation of a protruding bulk inside the cavernous cavity decreases its volume and compresses cavernosal tissue (23) and excessive folding may also lead to decreased distal rigidity (24).

For these reasons, we improved the technique of penile straightening in order to avoid disadvantages and to preserve the most important advantages of corporoplasty and tunical plication. The new technique avoids complete incision of the tunica albuginea which may compromise the erectile rigidity postoperatively (25) and at the same time, the new technique, gets the advantage of the healing edges and avoid the mere dependence on the suture as in plication techniques. Furthermore, in this new technique the superficial layers of tunica albuginea was ligated by absorbable suture covering the ethibond suture knots aiming to decrease the discomfort and/or pain caused the none-absorbable knots. However, in ventral curvature we preferred the 16 dot technique to avoid mobilization of neurovascular bundle and to maintain its integrity within the tunica albuginea. Also, we preferred the 16 dot technique for cases with Peyronie's disease to avoid incision and healing in these cases with idiopathic abnormal healing.

Results of group 2 were comparable to other study that used the 16-dot technique (11). Furthermore, the correction of penile curvature, residual curvature and recurrences, using the new technique, was comparable to another study that used corporoplasty (19).

In the present study, the post-operative complications, penile shortening and recurrence of penile curvature, were higher, with the 16-dot technique compared to the new technique but the differences were statistically insignificant. However, statistically significantly higher (P=0.005) post-operative complaint of feeling of a bothering palpable knot was reported by participants operated upon by the 16-dot plication technique. Furthermore, the post-operative erectile function was normally maintained in all participants as indicated by having a postoperative E4/E5, a non-significant difference between the pre and postoperative IIEF-5 scores and all participants retained their rigid erection during the whole period of follow-up. Those findings indicated the importance of using the new technique to correct the penile curvature.

## CONCLUSIONS

The new technique is superior to the 16 dot plication technique regarding post-operative feeling of a bothering suture knot especially when done for properly selected cases. It is better to be applied to cases with lateral or dorsal deviation of congenital penile curvature. Sixteen-dot technique is better to be applied to congenital ventral curvature and for patients with Peyronie's disease.
